# Observing mesoscale eddy effects on mode-water subduction and transport in the North Pacific

**DOI:** 10.1038/ncomms10505

**Published:** 2016-02-01

**Authors:** Lixiao Xu, Peiliang Li, Shang-Ping Xie, Qinyu Liu, Cong Liu, Wendian Gao

**Affiliations:** 1 Physical Oceanography Laboratory/Qingdao Collaborative Innovation Center of Marine Science and Technology, Ocean University of China, Qingdao 266100, China; 2 Scripps Institution of Oceanography, University of California San Diego, La Jolla, California 92093, USA

## Abstract

While modelling studies suggest that mesoscale eddies strengthen the subduction of mode waters, this eddy effect has never been observed in the field. Here we report results from a field campaign from March 2014 that captured the eddy effects on mode-water subduction south of the Kuroshio Extension east of Japan. The experiment deployed 17 Argo floats in an anticyclonic eddy (AC) with enhanced daily sampling. Analysis of over 3,000 hydrographic profiles following the AC reveals that potential vorticity and apparent oxygen utilization distributions are asymmetric outside the AC core, with enhanced subduction near the southeastern rim of the AC. There, the southward eddy flow advects newly ventilated mode water from the north into the main thermocline. Our results show that subduction by eddy lateral advection is comparable in magnitude to that by the mean flow—an effect that needs to be better represented in climate models.

## Abstract (web summary)

Physical processes by which mesoscale eddies affect subduction remain unclear. Here, through a specially designed eddy-resolving field experiment, the authors capture the eddy enhancement of mode water subduction and transport, and confirm the dominance of eddy lateral advection over the eddy trapping effect.

## Graphical abstract

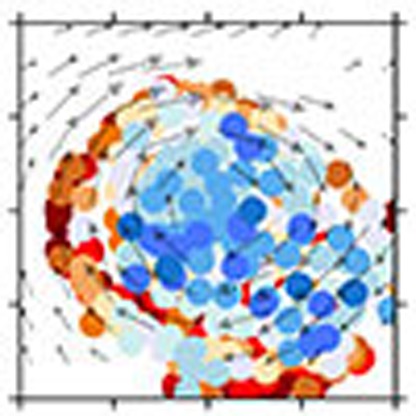

A universal feature of the global subtropical gyres is the presence of mode water, a thick layer of water with homogeneous properties within the main thermocline^1^,^2^ (Supplementary Note 1). Mode water is important in the climate system as ‘memories' of climate variability^3^ and by ‘breathing in' anthropogenic carbon dioxide^4^,^5^. Current climate models suffer large biases in representing mode-water subduction^6^,^7^. Mode-water subduction takes place near the western boundary current extensions with high eddy activities^8^,^9^. Previous studies propose the poor representation of mesoscale eddy effects as the major cause of the biases^7^,^10^. Results from eddy-resolving models suggest that mesoscale eddy contribution to the mode-water subduction is on the same order of magnitude as that by the mean flow^11^,^12^,^13^.

Physical processes by which mesoscale eddies affect subduction remain unclear, and are challenging to observe in the field. ACs have been observed to trap mode water while migrating equatorwards^14^,^15^,^16^,^17^; however, previous data did not resolve eddies to provide a quantitative estimate of eddy subduction and transport. The transport due to eddy-trapping is much weaker in the meridional than the zonal direction^18^, given the predominant westward propagation of mesoscale eddies^19^. In addition to the eddy-trapping transport, we propose a larger, complementary eddy lateral advective effect. Specifically, in the North Pacific, the southward eddy flow on the eastern rim of the AC advects south the dense, winter deep-mixed layer water from the north across large-scale density gradient into the permanent thermocline^7^. The cross-frontal eddy advection leads to an equatorward ‘bolus' transport^20^ of the newly ventilated mode water. Although this eddy-advective effect on mode-water subduction has been suggested from eddy-resolving model simulations^7^,^13^, it has never been verified in field observations.

The present study investigates this eddy lateral advective effect on subduction and transport of the North Pacific subtropical mode water (STMW) through a targeted field experiment: the Pacific Mode-Water Ventilation Experiment (P-MoVE; ref. 21) that made the first direct observations of eddy subduction processes in the western North Pacific. We deploy 17 Argo-profiling floats with enhanced daily sampling in an AC south of the Kuroshio Extension (KE) in late March 2014 (See Methods). On the basis of more than 3,000 hydrographic Argo profiles following the AC, we find that the southward eddy flow transports relatively low potential vorticity (PV) and apparent oxygen utilization (AOU) from the northern winter deep-mixed layer into the permanent thermocline, suggesting a ‘sweet spot' of subduction outside the eddy core on the southeastern rim of the AC. Together with results from the eddy-resolving model, we quantify that the southward eddy PV flux in the study region is comparable in magnitude to that by the mean flow, with the lateral advection dominating over the eddy-trapping effect. We conclude that the eddy lateral advection plays a key role in ventilating the interior of the upper ocean and increasing the net subduction of mode water.

## Results

### Observing eddy-induced subduction and transport

**Figure 1:**
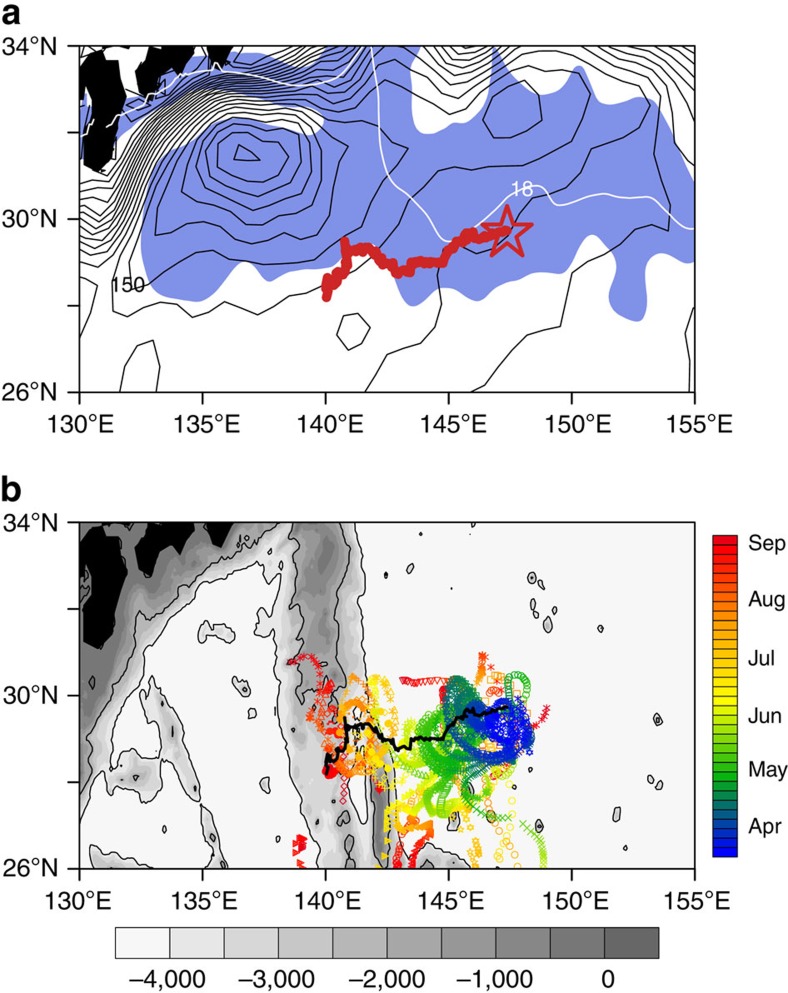
Trajectories of the AC and Argo-profiling floats. (**a**) Trajectory of the target AC centre from 27 March to 10 September 2014 (thick red line). The star sign denotes where the 17 Argo floats are deployed. The black contours denote the mean dynamical topography (contour interval (CI)=10 cm) to illustrate the large-scale circulation, and the blue shading highlights the STMW-formation region with SST of 17–19 °C (18 °C in white contour) on 27 March. (**b**) Trajectories of the 17 Argo floats (symbols with colours indicating the time). The trajectories end on 10 September 2014 when the AC dissipated. The symbol intervals for each float are daily. The grey shading indicates water depth (CI=500 m; the −2,000 and −4,000 m contours are highlighted in thin black lines). A major bathymetric feature in the region is the Izu Ridge along 140°E.

During the P-MoVE cruise, 17 Argo-profiling floats with enhanced daily sampling were deployed in an AC in the STMW-formation region in late March 2014 (Methods; Fig. 1a). The AC trajectory (red line in Fig. 1a) is determined by tracking the time-varying AC centre from satellite altimetry analysis, cross-checked against the geometry of velocity streamlines around the sea-level anomaly (SLA) maximum. Several Argo floats followed the westward-migrating AC through summer until the eddy disintegrated on encountering the Izu Ridge in late September (Fig. 1b). The floats provide more than 3,000 hydrographic profiles that offered an unprecedented detailed view of STMW subduction and dissipation. This section reports results from the field campaign.

**Figure 2:**
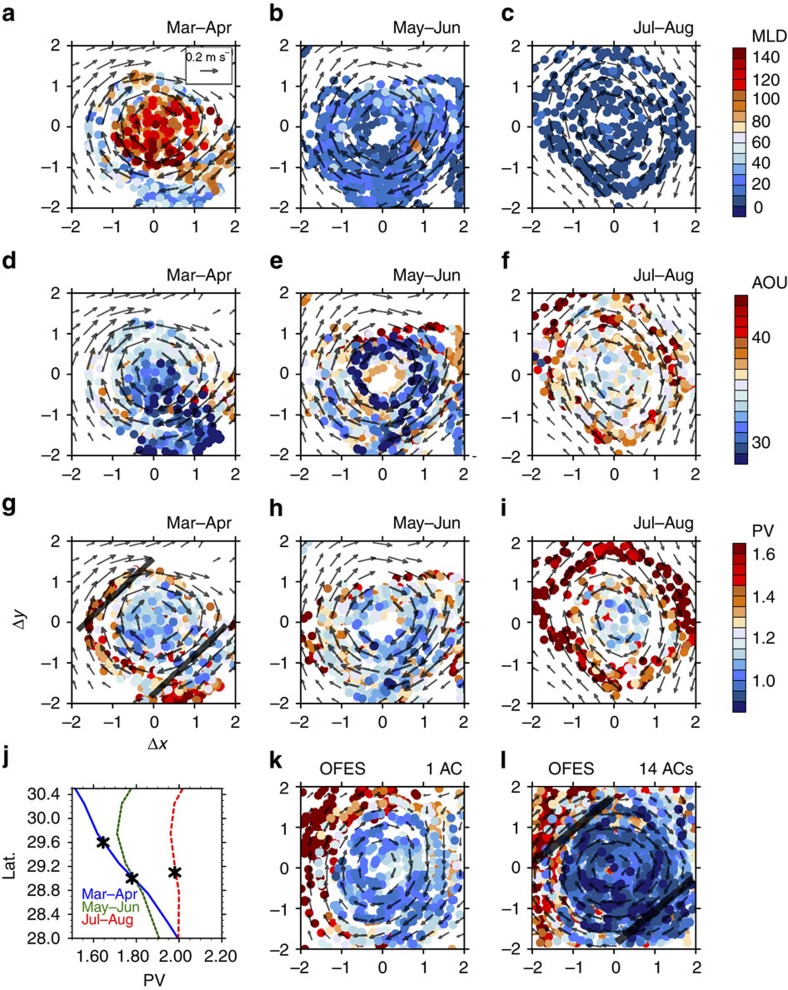
The AC fields in the core layer of STMW. Observed raw data dots are displayed in the top three rows: (**a**–**c**) mixed layer depth (MLD; m), (**d**–**f**) AOU (ml kg^−1^) and (**g**–**i**) PV (10^−10 ^m^−1 ^s^−1^) on the core layer of STMW (25.3*σ*_θ_). A coordinate system (Δ*x*, Δ*y*) is used relative to the AC centre (the red line in Fig. 1a or the black line in Fig. 1b). The outer boundary of the eddy core, defined as the zero relative vorticity contour, is normalized here as between [−1 1]. Arrows denote geostrophic currents in m s^−1^. The bottom left plot (**j**) denotes the zonally averaged (135°–150°E) PV on the core layer of STMW (25.3*σ*_θ_) as a function of latitude based on the Roemmich–Gilson Argo climatology: the solid blue line for March–April, dotted green line for May–June and dash red line for July–August. The latitude of AC is marked in black * in **j**. (**k**–**l**) Data dots of PV (colour shading) and geostrophic current (vectors) for March to April, sampled by synthetic Argo profiles deployed in OFES to mimic the sampling of the field campaign (Methods); (**k**) one AC in OFES and (**l**) 14 ACs in OFES. The slanted straight black lines in **g**,**l** are the positions of the transections in Figs 3 and 4.

**Figure 3:**
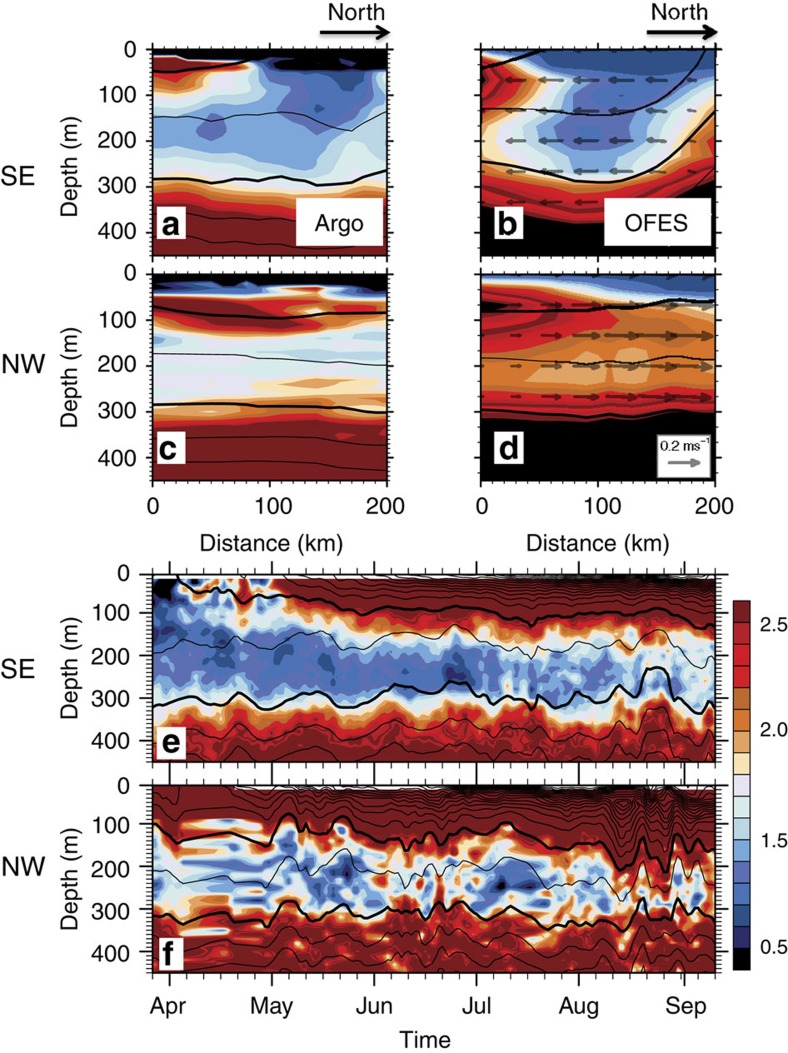
Vertical sections on southeastern and northwestern flanks of the AC. The southeastern and northwestern sections (thick black lines in Fig. 2g,l) are in the circumferential tangent direction of the AC. Vertical sections along (**a**,**b**) the southeast (SE) and (**c**,**d**) the northwest (NW) tracks for March to April, based on (**a**,**c**) the 17 Argo floats and (**b**,**d**) the eddy-resolving OFES (Methods). The horizontal ordinate is the distance from the southernmost point. The time-depth sections averaged along the (**e**) southeastern and (**f**) northwestern tracks are shown in the lower tow panels. Black contours denote potential density, thickened to highlight the 25.0 and 25.5 kg m^−3^ isopycnals that correspond to the top and bottom of the STMW layer, respectively. Coloured shades denote PV (10^−10 ^m^−1 ^s^−1^), and vectors in **b**,**d** show the along-track flow.

**Figure 4:**
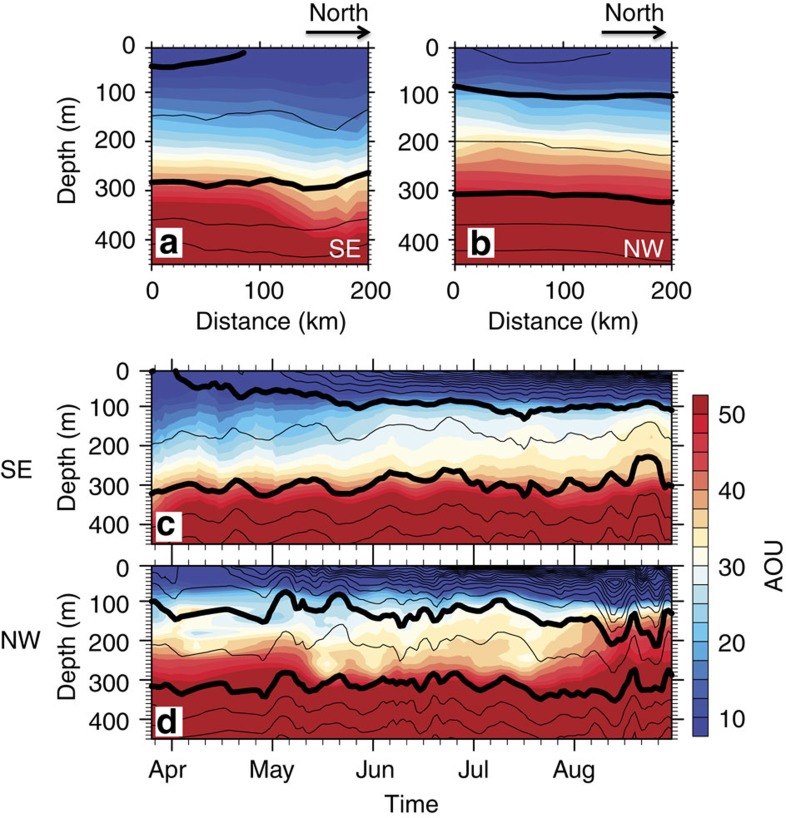
The AOU cross-sections along southeastern and northwestern flanks of the AC based on data from the 17 Argo floats. The southeastern and northwestern sections are denoted in thick black lines in Fig. 2g. Vertical sections along (**a**) the SE and (**b**) the NW flank of the AC during March to April; the horizontal ordinate is the distance from the southernmost point. Time-depth sections averaged along the (**c**) southeastern and (**d**) northwestern tracks. Colour shades denote AOU (ml kg^−1^). Black contours denote potential density fields; with thick curves represent the isopycnal 25.0 and 25.5 kg m^−3^, corresponding to the top and bottom of the STMW layer, respectively.

We use an eddy-following coordinate system—(Δ*x*, Δ*y*) for the relative position of the Argo floats to the AC centre—to construct the composite AC fields based on the data of the 17 Argo-profiling floats (Methods). Figure 2a–i shows in raw data dots the mixed layer depth, AOU and PV around the AC on the core density surface of STMW (25.3*σ*_θ_) for March to August. Here PV is calculated as 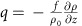, where *ρ* is potential density, *f* is the Coriolis parameter and *ρ*_0_ is a reference density (1024, kg m^−3^). Oxygen sensors have been deployed on the 17 Argo floats, and AOU is defined as the difference between the saturated and observed dissolved oxygen (DO) concentrations^22^. Similar patterns are obtained when the Argo data are interpolated on a 0.1 × 0.1 grid (Supplementary Fig. 1). During March–June, the data display east–west asymmetries (Fig. 2 and Supplementary Fig. 1), with larger mixed layer depth and lower values of PV and AOU in the southeast than in the northwest part of the AC (Supplementary Fig. 2a–c).We compare the vertical sections along the southeast and northwest tracks of the AC for PV (Fig. 3) and AOU (Fig. 4). The PV and AOU in the STMW layer show asymmetry between the southeast and northwest rims of the AC. Along the southeastern track of the AC, the STMW is ventilated and renewed with lowest PV (<1.5 × 10^−10 ^m^−1 ^s^−1^) and AOU (<25 ml kg^−1^) during March–April (Figs 3a,b and 4a). After earlier April, the STMW pycnostad loses contact with the atmosphere, but the PV (AOU) lower than 1.5 × 10^−10 ^m^−1 ^s^−1^ (25 ml kg^−1^) persists until June (Figs 3e and 4c), implying the eddy southward advection of low PV and AOU from the northern STMW-formation region. By contrast, on the northwest rim of the AC (Figs 3c,d,f and 4b,d), the PV and AOU in STMW are much higher (PV>1.5 × 10^−10 ^m^−1 ^s^−1^; AOU>35 ml kg^−1^). No ventilation occurs there, and the STMW pycnostad is sheltered from the surface even in March.

The subduction of low PV and AOU only happens on the southeast rim of the AC. There, the southward eddy flow carries low PV water from the deep-mixed layer into the permanent thermocline, suggesting a ‘sweet spot' of subduction by eddies (Figs 2a,g, 3a,b and 4a and Supplementary Fig. 1a,g). Owing to the southward intrusion of the deep-mixed layer and the thick mode-water layer underneath, the vertical density gradient is low on the eastern rim of the AC (Supplementary Fig. 3). In May–June, the STMW is capped by the seasonal thermocline that forms under surface warming (Fig. 3e). The southward advection of low PV and AOU (Figs 2e,h, 3e and 4c) by the eddy flow from the STMW-formation region in the north continues as the large-scale, meridional PV gradient remains strong (green dot line in Fig. 2j). From late June to August, by contrast, the newly formed STMW in the north erodes steadily as the seasonal thermocline develops, weakening the background meridional PV gradient (red dash line in Fig. 2j). The southward advection of STMW on the eastern rim of the AC weakens and eventually ceases (Fig. 2f,i).

Thus, our field observations have captured eddy effects on STMW subduction and ventilation. The PV asymmetry between the southeast and northwest rims of the AC (Figs 2g,h, 3e,f and Supplementary Fig. 2a–c) is a result of eddy advection across the background PV gradient. By advecting south the denser low PV water from the winter deep-mixed layer, the southward eddy flow acts to ventilate the interior of the upper ocean.

### Data model comparison

Does the current eddy-resolving model faithfully represent the observed eddy-advective effect on STMW subduction? To address this question, we deploy synthetic Argo floats in the Ocean General Circulation Model (OGCM) for the Earth Simulator (OFES) model, and use the ‘offline particle-tracking method' to track the floats. The Methods section (*Track ACs in the model*) describes how we deploy synthetic Argo floats in the eddy-resolving model OFES to mimic the sampling in the field. We tracked 14 ACs located in the study region (140°E–150°E, 28°–33°N) in late March. On the basis of the samples taken by the synthetic Argo floats, we construct the composite AC fields in OFES, and compare with observations.

We find that the eddy-resolving model successfully captures the eddy-advective effect. The east–west asymmetry in PV during March–April is confirmed in the OFES composites (Fig. 2k,l). As in observations, low (high) values of PV are found in the southeast (northwest) part of the AC (Supplementary Fig. 2d). The sampling of the east–west PV asymmetry can be improved by a more widely spread Argo array for the first few days; however, such an array is unstable on the outer rim of the eddy. In the model, the synthetic Argo floats away from the AC core cannot follow the AC movement and leave the AC soon.

**Figure 5:**
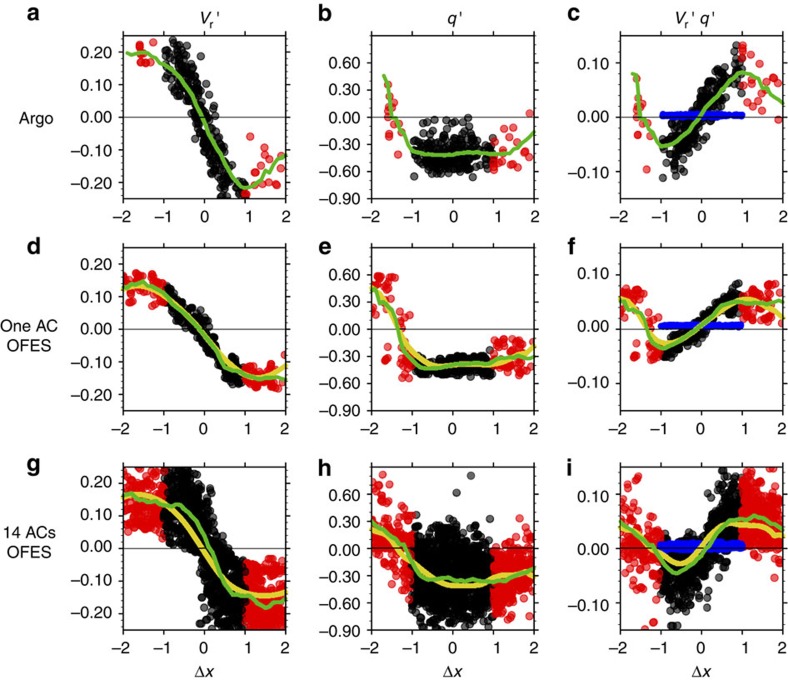
The meridional PV advection by ACs. The abscissa represents zonal sections across the AC centre. Recall that [−1, 1] defines the eddy core. (**a**,**d**,**g**) The tangential velocity of AC (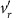 in m s^−1^); (**b**,**e**,**h**) the PV anomaly from the zonal-averaged background climatology (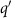 in 10^−10 ^m^−1^ s^−1^); and (**c**,**f**,**i**) the eddy-advective transport (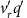 in black and red dots in 10^−10 ^s^−2^). The black dots are within the eddy core [−1, 1] where the integrated eddy flow transport is offset, and the red dots are in ±[1, 2]. The eddy-trapping transport (*C*_y_ in blue dots) is superimposed in the right panels. (**a**–**c**) for Argo observations, (**d**–**f**) for one AC and (**g**–**i**) for 14 ACs in OFES. Here we choose one AC from the model (**d**–**f**) that has the eddy PV flux close to that of the AC in the field to compare the zonal asymmetry of PV and tangential velocity. The green lines denote the average for each Δ*x*=0.1 bin calculated with the Argo sampling. The yellow lines in (**d**–**i**) are the exact calculation of the time average based on the full model data (Methods).

Figure 5 provides a closer look into the eddy-advective effect in both the model and observations. PV is nearly homogeneous inside the AC core that traps winter deep-mixed layer water from the AC origin, but is asymmetric outside the AC core between the west and east rims where the outer ring of the eddy stirs the ambient fluid. There, the southward (northward) eddy flow advects relatively low (high) PV into (from) the permanent thermocline (Figs 3a–d and 5a,b,d,e,g,h), with a net effect to strengthen the ventilation of the interior ocean. The majority of the eddy PV flux occurs outside the eddy core as detailed in the next section.

We chose an AC from the model that has the eddy PV flux close to that of the AC in the field to compare the zonal asymmetry of PV (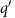) and tangential velocity (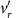) in Fig. 5. In OFES, the east–west PV asymmetry is sharper and the scatters of PV from the composite mean are narrower (Fig. 5e) than those in our observations (Fig. 5b), possibly because of insufficient horizontal dissipation^23^.

For comparison with the observed AC that trapped low PV water in the eddy centre (Fig. 2g–i), Fig. 2k,l shows results for ‘mode-water-trapping' ACs in the OFES model. Not all ACs trap low PV in their eddy centres (Supplementary Fig. 4a,b). However, both types of ACs show east–west PV asymmetry in the study region during March–April, illustrating the eddy lateral advective effect. Furthermore, cyclonic eddies (CCs) near the STMW ventilation region show similar eddy lateral advective effects (Supplementary Fig. 4c). The east–west PV asymmetry is comparable in magnitude between the two types of ACs and CCs (Supplementary Fig. 4d).

### Estimate the eddy-induced PV transport

On the basis of the Argo sampling, we estimate the time-averaged PV flux by a single AC during March and April on the core density surface of STMW, including the eddy-trapping and lateral advective transports (Table 1). The relationship between the eddy PV flux and the mode-water subduction rate is discussed in Methods. The Methods section (*Estimate the time-averaged PV transport by a single AC*) also describes in detail how we integrate the spatially and temporally varying Argo samples for the time-averaged eddy PV flux by individual ACs. We compare the estimates between the model and observations, and with the exact calculation from full model data (Fig. 5 and Table 1). The Argo sampling errors are less than 10%.

We compare the eddy-trapping and the eddy-advective PV transport based on the observed AC and the mode-water-trapping ACs we tracked in the model (Table 1). The net eddy-advective transport is large around the rim of the AC (1<|Δ*x*|<2), but it is almost zero within the eddy core ((|Δ*x*|<1), where the PV is well mixed and PV asymmetry is weak (Fig. 5c,f,i). The zonally integrated eddy PV flux by the observed AC with a normalized eddy radius is estimated at 8.24±0.82 × 10^−12 ^s^−2^ because of lateral advection, and 0.73±0.07 × 10^−12 ^s^−2^ because of the eddy-trapping effect. The eddy-trapping transport is smaller by one order of magnitude than the eddy lateral advective transport (Fig. 5c and Table 1). This result is confirmed by analysis of 14 ACs from the eddy-resolving model (Fig. 5i and Table 1).

The eddy lateral advective transport is comparable in magnitude to that by the mean flow. Directly calculated from OFES, the zonal-integrated PV transport on the core density surface of STMW across 30°N (140–150°E) by the mean flow is at 2.92 × 10^−6 ^m s^−2^, while the eddy-induced PV transport is at 2.88 × 10^−6 ^m s^−2^. Typically, there are 2.26 AC–CC pairs passing this section with an averaged eddy radius of 98.67 km. If we assume that eddy transport is symmetric between ACs and CCs, the equivalent PV flux by one single eddy (AC or CC) with a normalized eddy radius would be 6.45 × 10^−12 ^s^−2^. This is in broad agreement with the estimate by the synthetic Argo data in the model (7.09±0.71 × 10^−12 ^s^−2^). These results support the importance of the eddy lateral advection for STMW subduction.

We address the estimate errors associated with limited Argo samples by comparing the exact eddy PV transport based on the full model data with the estimates based on the synthetic Argo samples. We find that the sampling of 17 Argo profiles per day captures the sub-eddy-scale east–west PV asymmetry and the time-averaged meridional eddy PV transport during March and April (green and yellow lines in Fig. 5d–i). The error caused by the Argo sampling is less than 10%, compared with perfect sampling in the model (Supplementary Fig. 5). This lends some credibility to the estimates from our Argo observations. Although 17 Argo floats could not fully resolve the instantaneous eddy PV flux that varies in space and time (as indicated by scatters from the composite mean in Fig. 5), our enhanced daily sampling enables a time-averaged estimate with errors less than 10%.

Quantitatively, our observations of one single eddy captured eddy-advective effects in the fields but only allow for order of magnitude estimates of these effects. Our estimate of the eddy PV flux is subject to several sources of uncertainty due to the limited sample size (only one AC observed in the field) and spatiotemporal variability among mesoscale eddies. These uncertainties are difficult to quantify at the current stage. The eddy PV fluxes, especially the long-term mean, need to be better quantified in the future with more observations.

### Variations in mixing with the AC

**Figure 6:**
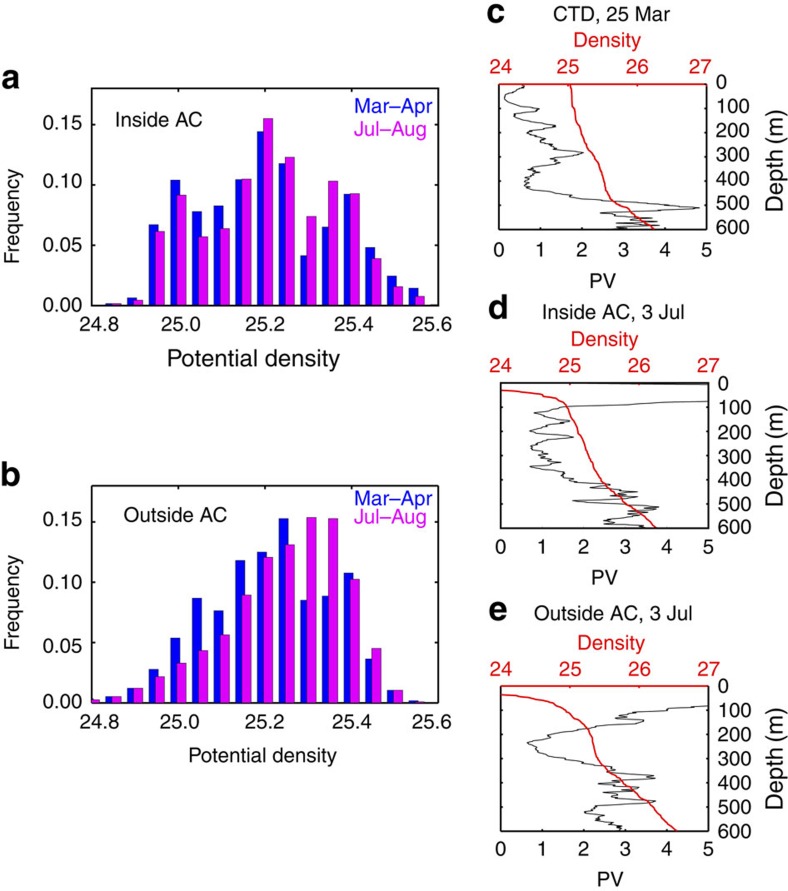
Seasonal variations of the multicore structure in and outside the AC core. The AC core is defined as the interior region enclosed by the maximum of Azimuthal speed. Proportion of profiles with vertical PV minimum in 0.05*σ*_θ_ bins based on Argo floats (**a**) in and (**b**) outside the AC core, with blue (magenta) bars for March–April (July–August). Results for May–June are similar to those for July–August. Vertical profiles of PV (10^−10 ^m^−1 ^s^−1^) and potential density (kg m^−3^) for (**c**) at CTD station at 148°E and 29.6°N on 25 March 2014, from Argo floats on 3 July, (**d**) in (World Meteorological Organization (WMO) ID 2901559) and (**e**) outside (WMO ID 2901563) the AC core. Vertical profiles in **d**,**e** are typical of multiple (single) core STMW in (outside) the AC in summer.

The isopycnal PV is well mixed within the AC core but displays a strong asymmetry outside the core in the east–west direction (Fig. 5). The enhanced temporal and vertical sampling of our Argo observations allows us to examine the difference in vertical mixing between inside and outside the AC core (Figs 2i and 6). As the season progresses, the low PV trapped in the AC core seems less dissipated than that in the surroundings (Fig. 2i), perhaps because the PV barrier reduces lateral mixing^18^. In many of our Argo profiles for March–May, the STMW displays a ‘multicore structure'^24^,^25^, with more than one PV minima in the vertical (Fig. 6c) both in and outside the AC core. Remarkably, the PV minima prefer to form at discrete densities of 25.0, 25.2 and 25.4*σ*_θ_ (Fig. 6a,b). In summer, pycnostads with multiple PV minima in STMW become rare outside the AC and gradually mixed into one single broad dense core (Fig. 6b,e). In the AC core, by contrast, the multicore structure of STMW persists even in summer (Fig. 6a,d), indicating a weak vertical mixing there. The summer histogram retains the characteristic tri-modal structure in the AC core, while it transforms into a smooth distribution outside that shifts towards higher density due to the seasonal erosion of the light STMW.

Our enhanced vertical sampling yielded a large number of high-resolution profiles that enables studying the seasonal evolution of the STMW multicore structure for the first time. Such studies will shed light on how the multicore structure forms and why it persists for a longer time inside the AC core.

## Discussion

We have observed the eddy-advective effect on mode-water subduction using a specially designed eddy-resolving array of 17 Argo-profiling floats deployed in an AC in the mode-water formation region. Our analysis of over 3,000 hydrographic profiles reveals that the PV and AOU are asymmetric about the AC centre, a structure indicative of lateral advection by the eddy. We further estimated that the southward eddy PV flux in the study region is comparable in magnitude with that by the mean flow, with the lateral advection dominating over the eddy-trapping effect. More eddy-resolving observations are needed to improve the estimates of long-term mean eddy fluxes. We found that the eddy lateral advection has significant seasonal variations, being strongest in winter and gradually weakening after June. The seasonal variations are closely related to the strength of the background PV gradient that forms between the newly ventilated STMW to the north and the old STMW to the south. As the seasonal thermocline develops, the background meridional PV gradient weakens, and the southward PV advection eventually ceases.

The maximum eddy subduction takes place around the eastern rim of the AC (1<Δ*x*<2; Figs 2g and 5). There, the subducted fluids may escape from the eddy into the interior ocean. Indeed, some of the floats left the eddy and moved southwards from the eastern rim of the AC (Fig. 1b). Besides, PV is not conservative along a trajectory of eddy flow as clear in the east–west asymmetry in Fig. 2g because of dissipation (for example, submesoscale processes). Thus, the eddy lateral advection causes a net PV flux to ventilate the interior of the upper ocean and to increase the net subduction of mode water. As long as meridional background PV gradients are maintained, the eddy advection is active, even if a fluid particle released in early winter circulates around the eddy and re-enters the mixed layer before the winter ends. This is different from Stommel's Ekman demon^26^.

While eddy tracer fluxes have been estimated with satellite altimetry and surface drifters^27^,^28^,^29^,^30^,^31^, previous studies mainly focused on the sea surface. Recent studies^32^,^33^ using subsurface Argo data investigated an important but different eddy effect of mixing high PV from polewards of the KE (a damping effect on mode-water formation). Our field campaign targets at the subduction process south of the KE. Our subsurface data resolve the AC in a statistical sense, permitting observational estimates of eddy subduction effects in the region for the first time.

**Figure 7:**
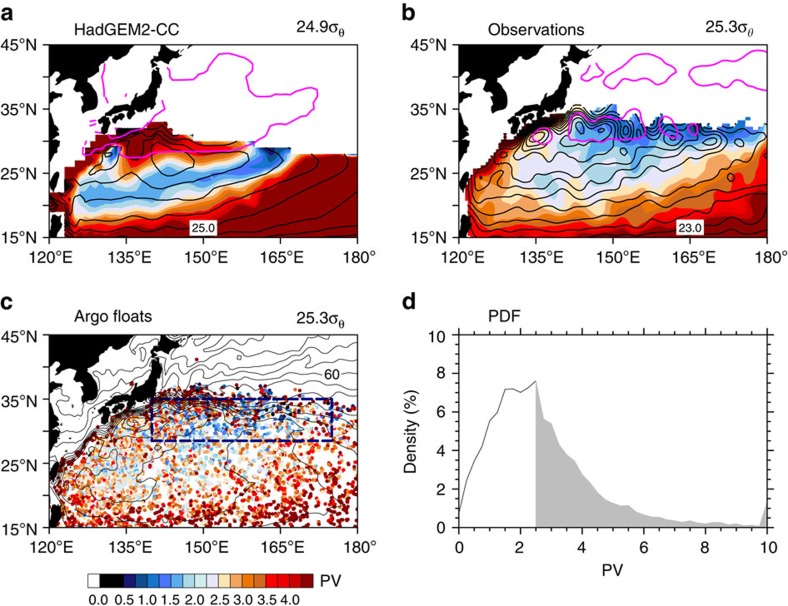
Isopycnal PV on the core layer of STMW in March for climate model and gridded and ungridded raw data of Argo. (**a**) A randomly selected CMIP5 climate model (HadGEM2-CC), where eddies are parameterized. (**b**) Gridded observations based on Argo-profiling floats; the streamlines (acceleration potential, CI=0.5 m^2^ s^−2^) are superimposed as black contours, and the winter MLD front (MLD=150 m contour) in thick magenta line. (**c**) The ungridded raw data of Argo, along with the mean dynamical topography (CI=10 cm). (**d**) PDF (%) of March isopycnal PV based on the Argo data for 2004–2014. Region for the calculation is denoted by the thick dashed rectangle in **c**. If we define the STMW using the critical PV value of 2.5 × 10^−10 ^m^−1 ^s^−1^, ∼49% of Argo-profiling floats do not contain STMW in its formation region in March (grey shading in **d**).

Eddy advection significantly affects the subduction and transport of STMW. In coarse-resolution climate models (Fig. 7a), the subduction of low PV water is confined to a narrow point, and the southward transport of low PV is along the mean streamline (Fig. 7a), indicating the dominance of the mean flow advection. By contrast, in gridded Argo observations, STMW subducts into the thermocline in a broad region of the winter deep-mixed layer (Fig. 7b). After subduction, the PV minimum is trapped near the outcrop within a narrow band. The width of the low PV band is ∼200–300 km, corresponding to the length scale of mesoscale eddies. Even within the climatological low PV band, ∼49% of historical Argo profiles do not contain STMW (Fig. 7c,d and Supplementary Fig. 6), another fact that underlines the importance of eddies for the subduction and transport of STMW.

A popular parameterization of geostrophic eddies widely used in climate models is the Gent and McWilliams scheme^34^,^35^. Coupled Model Intercomparison Project Phase 5 (CMIP5)-class models typically use Gent and McWilliams diffusion coefficients less than 1,000 m^2^ s^−1^ (refs 36, 37). The values seem to be too small. Our Argo observations imply an eddy diffusivity on the order of (10^4 ^m^2 ^s^−1^) in the study region (see Methods). This is consistent with several recent modelling studies that have advocated increasing the near-surface diffusivity to several thousand m^2 ^s^−1^, but with a regional structure that peaks at O (10^4 ^m^2 ^s^−1^) in the most energetic area of the ocean^10^.

The value of our study is in the field campaign that was designed to test the hypothesis that the lateral advection by eddies enhances mode-water subduction. To our knowledge, it is the first time that this eddy effect has been detected in the field. We have successfully deployed an eddy-resolving array of 17 Argo floats inside an AC. Setting the parking depth at 500 dbar helped to keep many Argo floats trapped within the eddy, and the enhanced daily sampling yielded enough data for eddy-resolving composites. Reduced temporal sampling sharply reduces the number of profiles on the AC rim, deteriorating the representation of the sub-eddy-scale east–west asymmetry in PV structure (Supplementary Fig. 7). The enhanced vertical sampling, on the other hand, enables a large-scale study of seasonal evolution of the STMW multicore structure, a project that is underway. Additional eddy-resolving field experiments are necessary to better quantify the large-scale effects of mesoscale eddies.

The KE is the window where the winter-mixed layer ventilates the main thermocline of the North Pacific to the south. The ventilation makes the KE an important carbon sink^38^,^39^,^40^. Results from our field campaign call for improved representation of eddy effects on the subduction and transport of STMW to achieve more realistic simulations of the regional biogeochemical cycle and reliable projections of its change.

## Methods

### The field experiment

To investigate the eddy effect on subduction and transport of the STMW, the P-MoVE cruise deployed 17 Argo-profiling floats (World Meteorological Organization IDs 2901550–2901566) inside an AC. We considered ACs located west of 150°E (A and B in Supplementary Fig. 8) in late March 2014 for deployment. Since the AC A was close to the Izu Ridge, it might dissipate over the rough topography. Thus, we chose the AC B near 147.5°E, 29.5°N, which is a typical eddy with a radius of 71.5 km and rotational speed of 29 cm s^−1^ (Supplementary Fig. 9).

The P-MoVE was conducted on the Research Vessel (R/V) *Dongfanghong 2* from 17 March to 23 April 2014. During the cruise, the target AC position was identified in advance from the near-real-time SLA field and communicated to the vessel each day. Conductivity, temperature, and depth (CTD), expendable bathythermograph (XBT) and shipboard acoustic Doppler current Profiler measurements (Supplementary Fig. 10a) were first conducted around the target AC to identify the AC centre before deploying the Argo-profiling floats. A significant difference in the vertical temperature profiles existed between the western (black line in Supplementary Fig. 10b) and eastern (red line in Supplementary Fig. 10b) sides of the AC. Vertical temperature gradient was much weaker on the eastern side of the AC, suggesting fresh STMW formation there. Seventeen Argo floats were deployed on 27 March 2014 inside the AC (see deployment locations in Supplementary Fig. 10c). We deployed the Argo floats with an emphasis on the southeast part of the AC.

To keep the floats trapped in the migrating AC, we set the parking depth at 500 dbar. The floats dive to 1,000 dbar from the parking depth before the ascent, during which they conduct temperature, salinity and DO measurements. Each float makes observations daily as it rises from 1,000 dbar to near the sea surface, with a 2-dbar sampling interval above 600 m and 10 dbar below it. Compared with the conventional Argo sampling intervals of 10 days and 10 dbar, our enhanced time sampling proves crucial for our Argo array to maintain eddy-resolving capability, while the enhanced vertical sampling is instrumental in identifying reduced mixing of mode water within the AC core as detailed in Results.

### Other data

To complement the P-MoVE Argo data, we include the following data: the historical ungridded raw data of Argo in the domain of interest (15–45°N, 120°–180°E) from 2004 to 2014, the daily SLA data from AVISO^41^ on a horizontal grid of 1/3°, the eddy-tracking data set of Chelton *et al.*^19^ and the daily outputs from OFES with a horizontal grid of 1/10° (ref. 42). All the Argo data passed the Argo real-time quality control, and those flagged as bad are eliminated. For the comparison of observational and simulated quantities, we use the gridded monthly Roemmich–Gilson Argo Climatology^43^, the mean dynamical topography from the CNES-CLS09 product^44^ and a randomly selected climate model—HadGEM2-CC from CMIP5 under the historical scenario.

### Composite AC fields

On the basis of the daily SLA data from AVISO and the hydrographic profiles of the 17 Argo-profiling floats, we construct composites referenced to the AC centre (the red line in Fig. 1a or the black line in Fig. 1b). An eddy-following coordinate system (Δ*x*, Δ*y*) is used for the relative position of the Argo floats to the AC centre. The each-day AC centre is first identified based on the geometry of velocity streamlines around the SLA maximum. We interpolate each T–S and oxygen profile vertically on 1-dbar intervals using the Akima spline. Argo DO profile data are checked with shipboard CTDO and DO in the water samples at the time of launch (not shown here). Argo DO measurements failed the global range test after August 2014. The DO data after that are excluded.

### Track ACs in the model

On the basis of the daily outputs of OFES, we use the ‘offline particle-tracking' method to deploy synthetic Argo floats to track ACs in the model. We choose 14 ACs located within 140°E–150°E, 28°–33°N in March for 1994–2007. Seventeen synthetic Argo floats were deployed for each AC, and the deployment locations are similar as in Supplementary Fig. 10c. In line with the parking depth of 500 m, the synthetic Argo float's movement was determined by the model's daily velocity at 500 m. We record the float trajectory every day and take a vertical profile. Since the target AC is a ‘mode-water eddy' where low PV water is trapped in the AC centre, we only track ACs with a low PV core in the model for one-to-one data-model comparison.

### Estimate the time-averaged PV transport by a single AC

We define the time-averaged meridional PV (*q*) transport on the core density surface of STMW, 25.3*σ*_θ_, across a zonal section (−2<*x*<2; the unit is the normalized distance from the eddy centre to the outer boundary of the eddy core) that the AC occupies as:


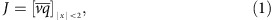


where the bracket denotes zonal integration and the over bar denotes time mean. Note that equation (1) is under the eddy-following coordinate system. We can divide *v* (the meridional flow speed) and *q* into the climatological zonal average and the deviation from the climatological zonal average, 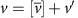 and 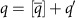. Then, the time mean meridional PV transport is 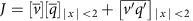. We further define the eddy-induced velocity as:


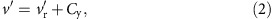


where 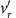 is the relative velocity to the moving frame and *C*_y_ is the AC propagation velocity. For the ‘mode-water-trapping' AC (Fig. 5), *C*_y_ is constant but 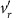 is wholly antisymmetric about the AC centre, while 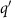 is well mixed within the eddy core (|Δ*x*|<1) but antisymmetric around the rim of the AC (1<|Δ*x*|<2). Thus, 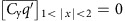 and 
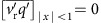
. The total meridional transport can be decomposited as follows:


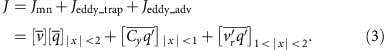


Here the eddy effects are divided into two parts: the Eddy-trapping effect 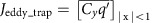 and the non-eddy-trapping lateral advective effect 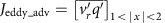.

Using equation (3), we estimate the time-averaged eddy PV flux for March–April based on the Argo sampling. We first calculate 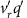 (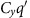) directly for each Argo sample (−0.5<*y*<0.5), and then obtain the time mean 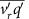 per Δ*x*=0.1 bin (solid green line in Fig. 5), and finally integrate 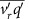 zonally for the total eddy PV flux by one AC (Table 1).

This estimate (here after 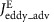) contains errors due to limited Argo sampling. To quantify the error, we compare the estimate 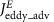 to the exact calculation (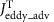) from the full model data. For the full model data, we first calculate the instantaneous 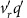 (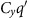) for each grid point around the AC, and then integrate temporally and zonally. We calculate the estimate error, 
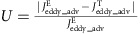
, for each AC we tracked in OFES and find that the error due to limited Argo sampling is less than 10% (Supplementary Fig. 5). Including the ±10% error due to limited Argo sampling, we add a value range for the estimate of the eddy PV flux in Table 1.

### Relation between eddy PV flux and subduction rate

The meridional PV flux (*T*) on the core density surface of STMW is the product of velocity (*v*) and PV (*q*),


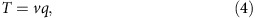


and the time mean PV flux is


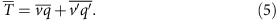


The time mean PV transport can be rewritten as a ‘transport velocity' after dividing by the mean PV,


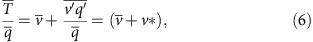


where the eddy-induced velocity in equations (6) is 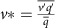, known as the ‘bolus velocity'^20^.

Following Marshall^11^, the water mass subduction rate (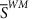) in an eddying ocean can be described in terms of transport velocities


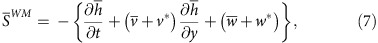


where [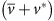, 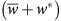] is the transport velocity at the base of the mixed layer, *z*=−*h*. In particular, the subduction by mesoscale eddies is


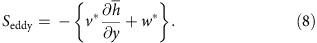


From equations (6) and (7), we obtain the relation between the subduction rate and PV flux that transports low PV water from mixed layer into the permanent thermocline:


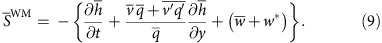


The comparison between the mean and the eddy PV flux in the present study reflects the relative contribution of the mean and the eddy flow to the subduction rate by lateral induction.

### Estimate the eddy diffusivity coefficients

According to previous studies^6^,^35^, the eddy PV flux is related to the mean PV gradient by an eddy closure hypothesis,


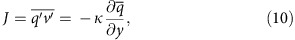


where *κ* is the eddy diffusivity. Here we use equation (10) to diagnose *κ*.

The averaged eddy PV flux for the target AC 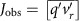 is estimated at 8.24±0.82 × 10^−12 ^s^−2^ (Table 1). This calculation for zonal integration is within [−2*R*, 2*R*], where *R* is the eddy radius. As the target AC is a typical eddy in the study region (Supplementary Fig. 9), we extend the estimate to a broad region by considering the frequency of eddy occurrence


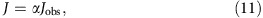


where *α* is the frequency of the eddy occurrence in the study region (135–175°E, 28–32°N). Here *α* is defined as:


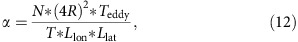


where *T* is the total length of observations, *L*_lon_ and *L*_lot_ are the zonal and meridional distances of the study region, respectively, *N* is the total number of mesoscale eddies (both ACs and CCs, if we assume that eddy PV transport is symmetric between them), 4*R* represents the extent for integration for *J*_obs_ and *T*_eddy_ is the averaged lifetime of mesoscale eddies.

On the basis of the eddy-tracking data set of Chelton *et al.*^19^ for 1992–2012, in the study region (135–175°E, 28–32°N), the total number of mesoscale eddies *N*=1,147 (eddies near the boundaries are excluded), the average eddy radius *R*=89.5 km and the averaged lifetime of mesoscale eddies *T*_eddy_=11 weeks. Together with the length of observations *T*=1,095 weeks, and the zonal and meridional distances of the study region *L*_lon_=3,849.3 km and *L*_lat_=444.5 km, respectively, *α* is estimated as 86.3%. We obtain a long-term mean eddy PV flux in the study region, *J*=7.11±0.71 × 10^−12 ^s^−2^.

The corresponding mean PV gradient 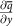 is estimated to be −1.64 × 10^−16 ^m^−2 ^s^−1^ based on the Roemmich–Gilson Argo climatology (blue line in Fig. 2j). Correspondingly, the eddy diffusivity is in the range of (4.34±0.43) × 10^4 ^m^2 ^s^−1^. Our Argo observations imply an eddy diffusivity on the order of 10^4 ^m^2 ^s^−1^ in the study region.

## Additional information

**How to cite this article:** Xu, L. *et al.* Observing mesoscale eddy effects on mode-water subduction and transport in the North Pacific. *Nat. Commun.* 7:10505 doi: 10.1038/ncomms10505 (2016).

## Supplementary Material

Supplementary Information Supplementary Figures 1-10, Supplementary Note 1 and Supplementary References.

## Figures and Tables

**Table 1:** The time-averaged meridional PV transport by a single eddy during March and April.

	**Lateral advective transport**	**Trapping transport**
Argo observations	8.24±0.82	0.73±0.07
OFES	7.09±0.71	0.72±0.07

AC, anticyclonic eddy; OFES, OGCM for the Earth Simulator; PV, potential vorticity.

On the basis of the Argo sampling (Methods), the eddy lateral advective and trapping transports (in 10^−12 ^s^−2^) are estimated for the observed AC, and the average of 14 ACs in OFES (Fig. 5i). The range of value includes the ±10% error due to limited Argo sampling.
