# Supplementary material for: Observing mesoscale eddy effects on mode-water subduction and transport in the North Pacific
Source: Nat Commun. 2016 Feb 1;7:10505. doi: 10.1038/ncomms10505 (PMC4740428; doi:10.1038/ncomms10505)
Supplement: Supplementary Information — Supplementary Figures 1-10, Supplementary Note 1 and Supplementary References. [file ncomms10505-s1.pdf]

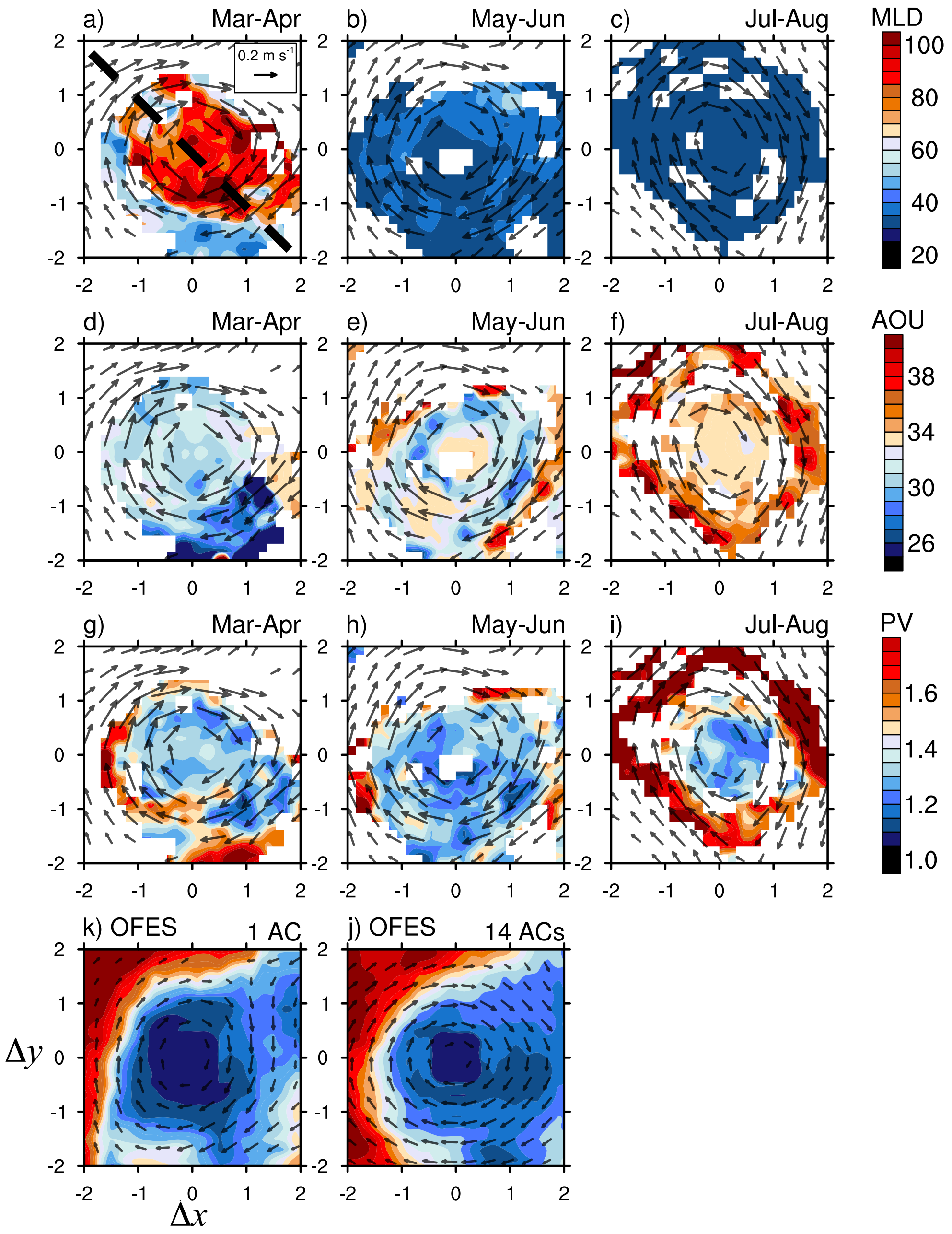

Supplementary Figure 1 | Gridded isopycnal AC fields. a-c) MLD (color shade in m), d-f) AOU (color shade in ml kg^{-1}), and g-i) PV (color shade in 10^{-10} m^{-1} s^{-1}) based on the Argo samples**.** The gridded PV field based on k) one AC, and j) 14 ACs in OFES. Vectors are the geographic currents in m s^{-1}**.** The black dash line in a) is the position of transaction shown in Supplementary Fig. 2.

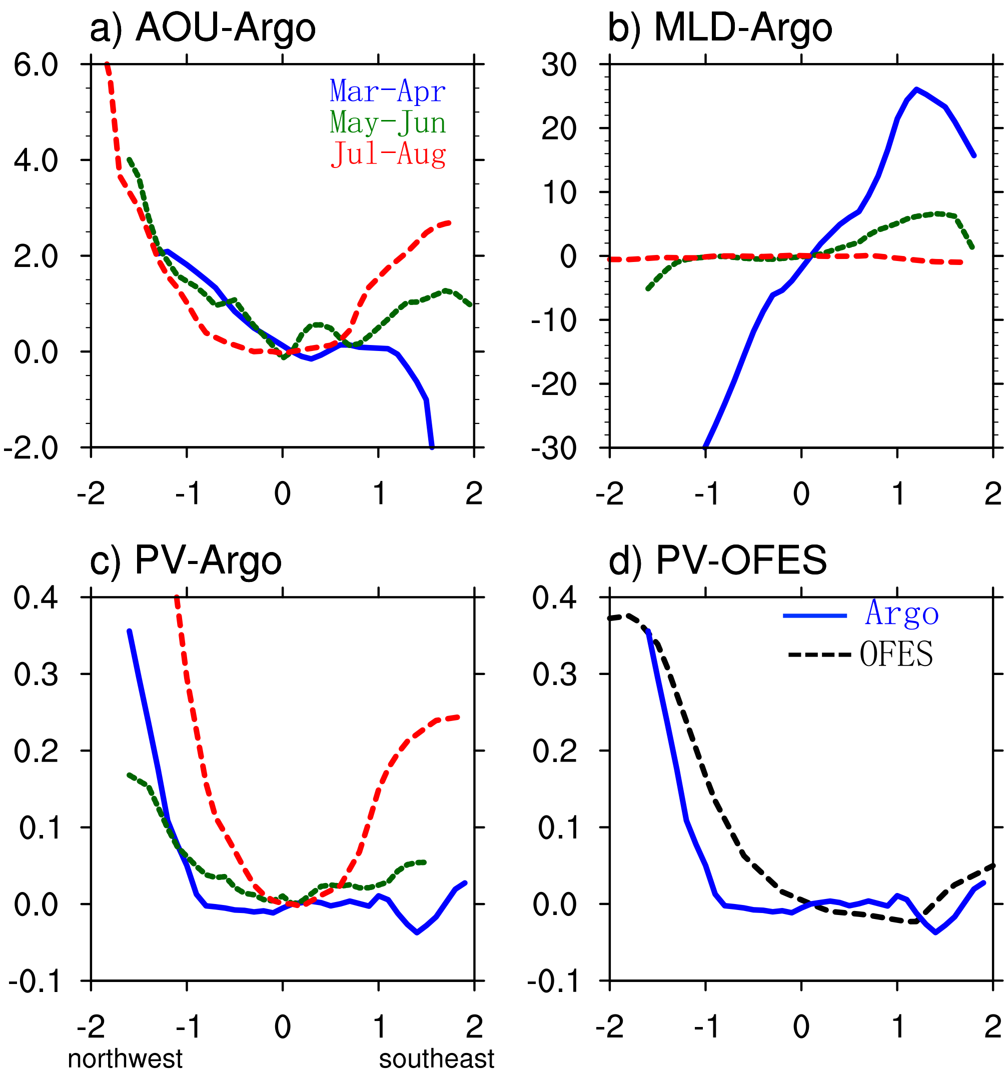

Supplementary Figure 2 | Asymmetry features around the AC center. The abscissa represents the northwest-to-southeast section (dash black line in Supplementary Fig. 1a). The ordinate represents difference of a) AOU (ml kg^{-1}), b) MLD (m), and c-d) PV (10^{-10} m^{-1} s^{-1}) from the AC center. a-c) are based on the Argo samples; the solid blue line for March-April, dotted green line for May-June and dash red line for July-August. The bottom right plot d) is a comparison of PV for March-April between the Argo data (solid blue line) and the eddy resolving model OFES (dash black line).

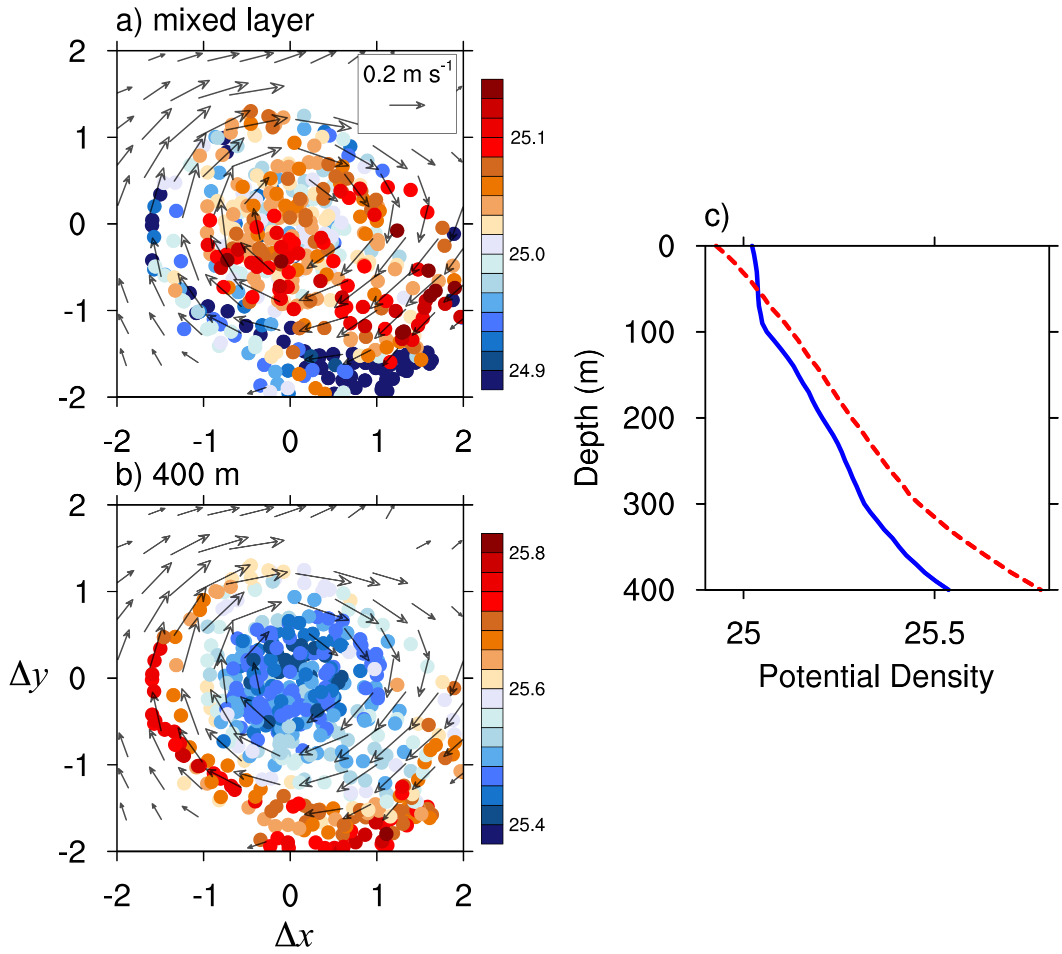

Supplementary Figure 3 | The asymmetry of vertical stratification. The observed data dots of potential density (kg m^{-3}) at a) the mixed layer and b) 400 m depth for March-April. Similar as in Figs. 2a-i, arrows denote geostrophic currents in m s^{-1}, and the range of [-1, 1] represents the outer boundary of the eddy core. Note different colorbar for a-b. c) The vertical profile of potential density on the eastern (western) flank of the AC is shown in solid blue (red dashed) line.

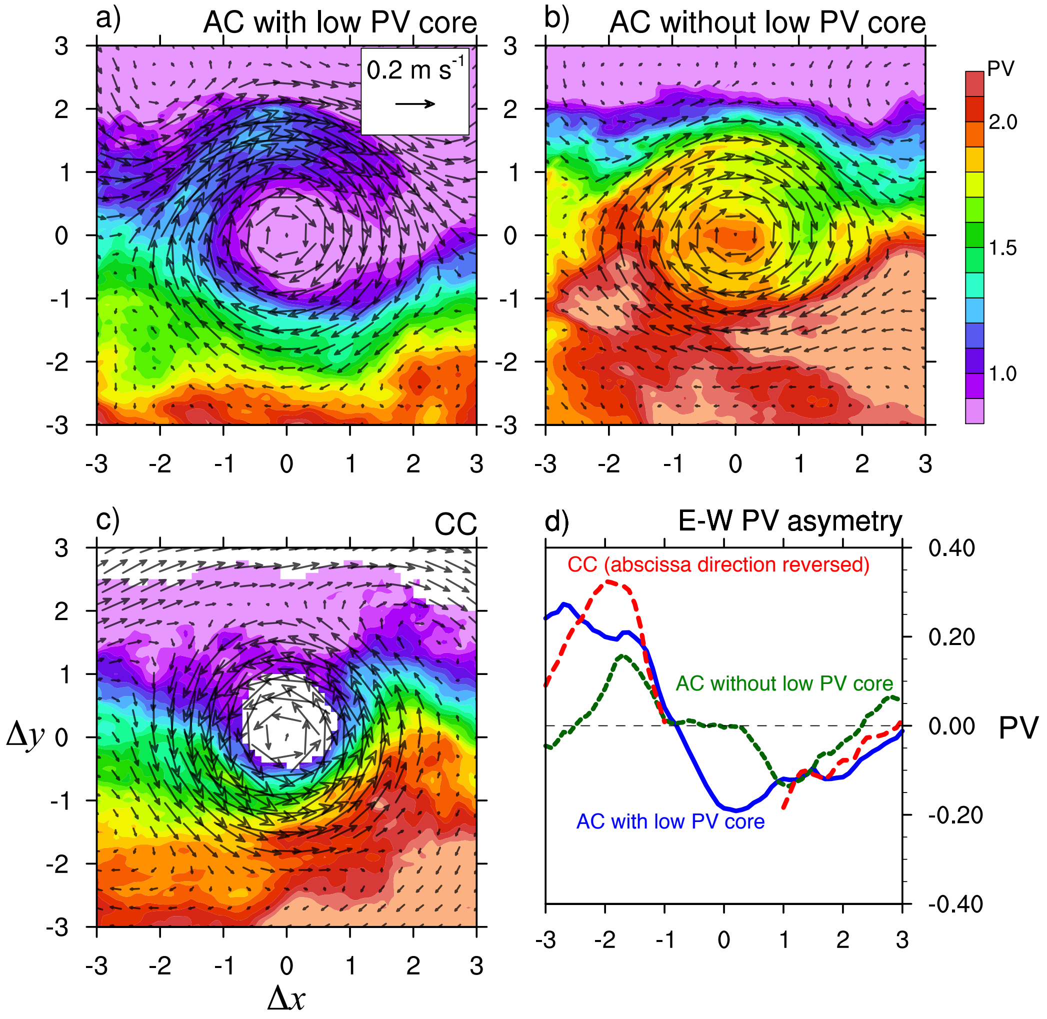

Supplementary Figure 4 | Composite of PV and velocity fields around eddies in the study region based on the full model data of OFES. Anticyclonic eddies a) with low PV water and b) without low PV water in the eddy center, and c) cyclonic eddies. PV is denoted by color shading (10^{-10} m^{-1} s^{-1}), while velocity is by vectors (m s^{-1}). d) The east-west asymmetry of the PV anomaly from the zonal averaged background climatology (in 10^{-10} m^{-1} s^{-1}) based on a-c); Note we reverse the abscissa direction for the case of cyclonic eddies (red dash line in d). The composite is in 28°-31°N, 130°-180°E.

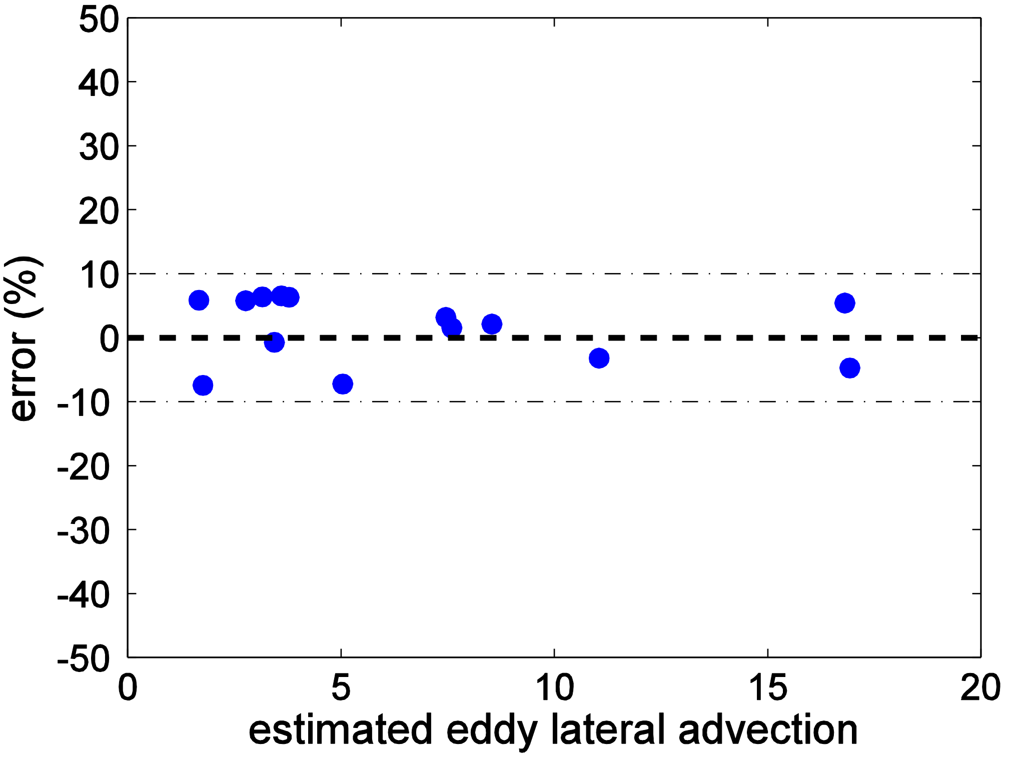

Supplementary Figure 5 | Errors in the estimate of eddy lateral PV advection due to limited Argo sampling. Blue dots represent results from 14 ACs in OFES**.** The abscissa represents the estimate of eddy lateral advection by synthetic Argo sampling (in 10^{-12} s^{-2}). The ordinate represents errors (%) due to limited sampling (Methods). Error level of $\pm$10 % is superimposed in dash dotted lines, suggesting the upper and lower bound of the estimate errors.

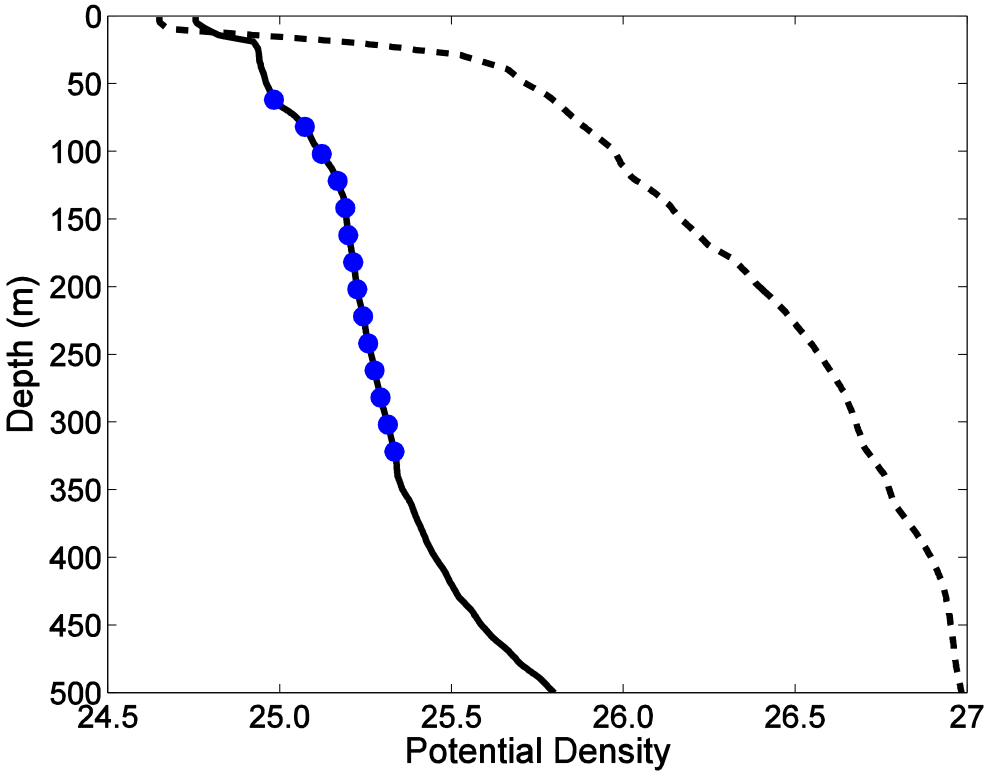

Supplementary Figure 6 | Comparison between two snapshot Argo profiles. Both near 150.0°E, 31.0°N, one with mode water (May 22th, 2005; solid line) and the other without (May 13th, 2009; dashed line). Blue dots denote the mode water layer, which appears as a minimum in the vertical gradient of density.

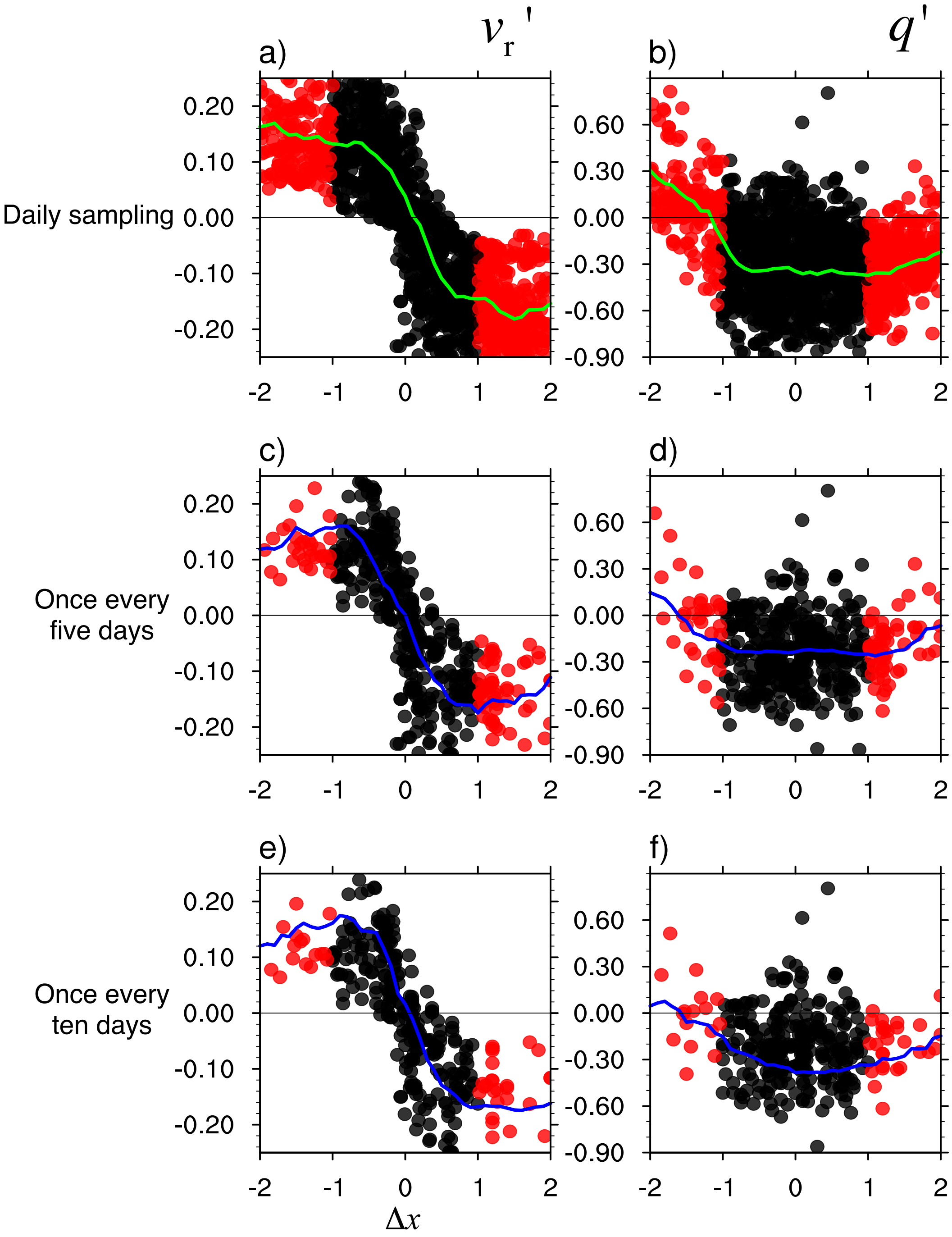

Supplementary Figure 7 | Blurred PV asymmetry when the sampling intervals of the 17 synthetic Argo floats reduce. The sampling intervals change from a-b) daily to c-d) once every five days, and e-f) once every ten days. a-b) are the same as Figs. 5 g-h. The solid green and blue lines denote the average for each Δx = 0.1 bin. The results are based on the 14 ACs we tracked in OFES.

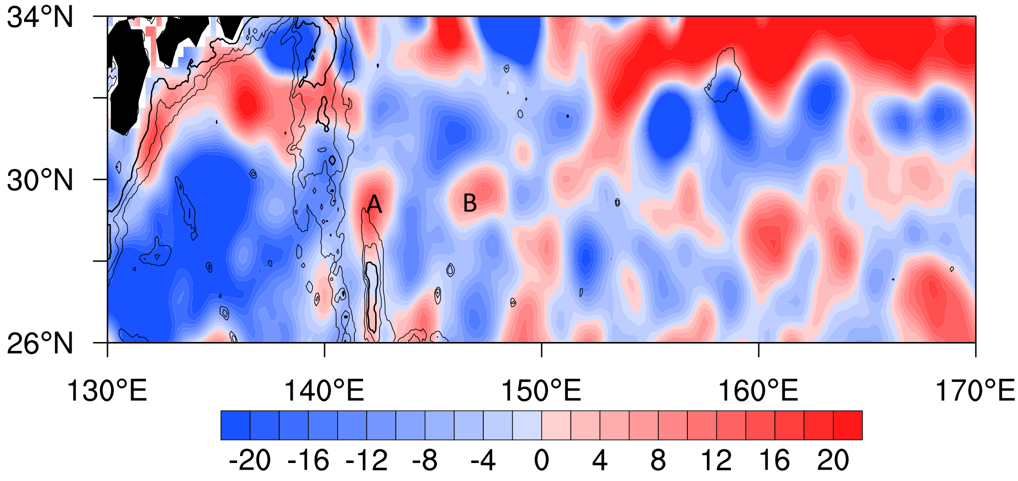

Supplemenary Figure 8 | Near real time SLA on 27 March 2014. ACs to the west of 150°E are labeled as A and B (the target AC). The SLA is denoted by color shade in cm. Black contours denote -3000 m, -2000 m and -1000 m (bold line) depth of water. Major bathymetric features in the region include the Izu Ridge along 140°E and the Shatsky Rise around 159°E.

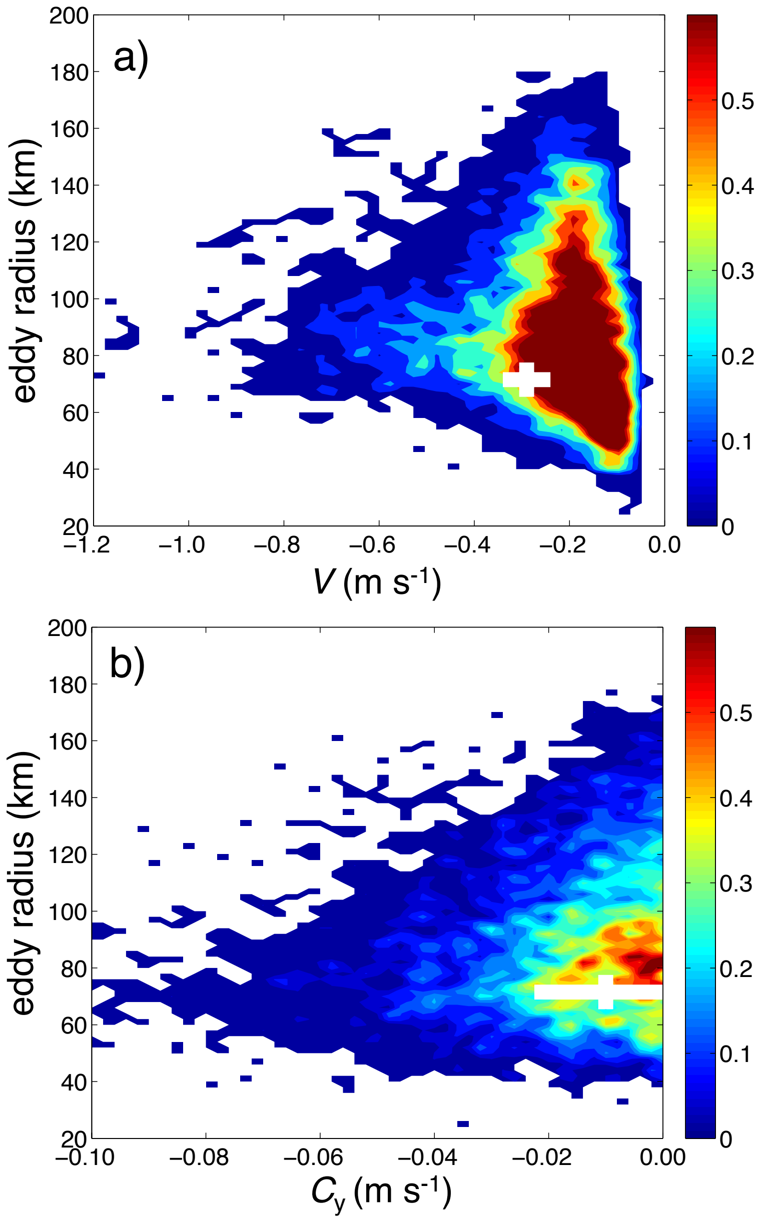

Supplementary Figure 9 | Statistics of the ACs in the study region. We investigated the eddy statistics in the study region (135ºE-175ºE, 28ºN-32ºN) based on the eddy tracking dataset of Chelton et al. Probability density (shading in %) for the ACs from 1992 to 2012 as a function of a) southward rotational eddy flow (*v*) and eddy radius, and of b) eddy propagation speed (C_y) and eddy radius. The white plus denotes features of the target AC, and the length of the line denotes the range of variability (one standard deviation).

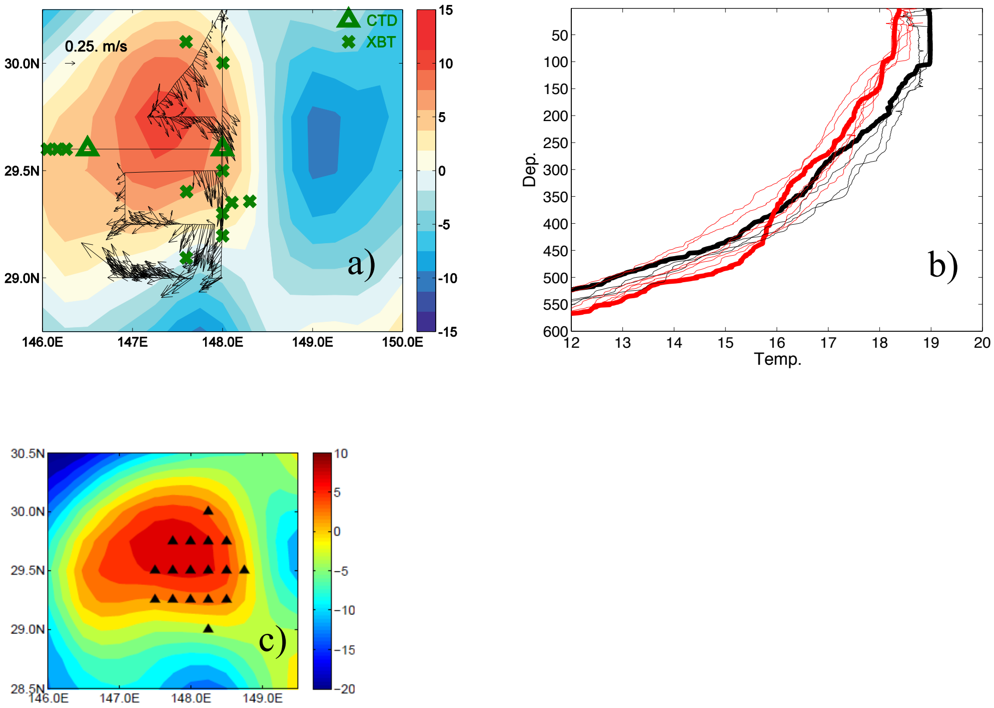

Supplementary Figure 10 | Field observations around the target AC. a) Vectors show the ADCP measured ocean currents at 100 m depth. Thin black line denotes the ship routing. The XBT and CTD stations are shown in green x and triangle, respectively. Color shading is the SLA field on 27 March 2014. b) Temperature profiles from CTD (thick line) and XBT (thin line). CTD profiles on the east (west) side of the AC are shown in red (black). c) Deployment stations of the 17 Argo floats are shown in black triangles, and color shading is SLA on 27 March 2014.

Supplementary Note 1 ── Introduction to Mode Waters

The subtropical mode waters (STMW) appear as a minimum in the vertical gradient of temperature and density between the seasonal and permanent thermocline. They are formed in the deep mixed layer on the warm side of the strong Kuroshio Extension current in late winter, capped by the seasonal thermocline through the succeeding surface warming, and widely distributed in the subtropical gyre by ocean circulation[1-3]. In association with the formation and circulation of STMW, temperature, salinity, oxygen, and PV anomalies are subducted downward to the subsurface layer. Moreover, they are observed to play an important role in the southward spreading of the Fukushima-derived radiocesium[4] and the nutrient cycling in the oligotrophic subtropical gyres[5].

The accumulation of the Argo profiling floats captures more detailed structures of STMW. Based on the un-gridded raw data of Argo floats, the formation and distribution of the STMW in the North Pacific are found to be highly variable in space and time. Even near the outcrop of STMW where exists the climatological minimum PV (Fig. 7c), about 50% of the Argo profiling floats could not detect STMW (Fig. 7d).

In a fixed point (150.0°E, 31.0°N) within the region of the minimum PV band, the STMW can only be observed occasionally (Supplementary Fig. 6).

Supplementary References

1. Stommel, H. Determination of water mass properties of water pumped down from the Ekman layer to the geostrophic flow below. *Proc. Natl. Acad. Sci. USA*, **76,** 3051-3055 (1979).
2. Xie, S.-P., Kunitani, T., Kubokawa, A., Nonaka, M. & Hosoda, S. Interdecadal thermocline variability in the North Pacific for 1958-1997: A GCM simulation. *J. Phys. Oceanogr.* **30,** 2798-2813 (2000).
3. Suga, T., Aoki, Y., Saito, H. & Hanawa, K. Ventilation of the North Pacific subtropical pycnocline and mode water formation. *Prog. Oceanogr.* **77,** 285-297 (2008).
4. Kumamoto, Y. et al. Southward spreading of the Fukushima-derived radiocesium across the Kuroshio Extension in the North Pacific. *Sci. Rep.* **4,** 1-9 (2014).
5. Palter J. B., Lozier M. S. & Barber R. T. The effect of advection on the nutrient reservoir in the North Atlantic subtropical gyre. *Nature* **437,** 687-692 (2005).
